# Procedural Optimization of Capsaicin 8% Patch Therapy Using Ultrasound-Guided Bilateral Ankle Nerve Block in Painful Diabetic Peripheral Neuropathy: A Retrospective Comparative Cohort Study

**DOI:** 10.3390/healthcare14162563

**Published:** 2026-08-16

**Authors:** Hassan Moria

**Affiliations:** Department of Surgery, Faculty of Medicine, University of Tabuk, Tabuk 71491, Saudi Arabia; hmoria@ut.edu.sa; Tel.: +966-543474342

**Keywords:** diabetic peripheral neuropathy, capsaicin, regional anesthesia, ultrasound-guided nerve block, procedural analgesia, patient-reported outcomes, rescue analgesia, multimodal analgesia, ambulatory care, pain management

## Abstract

**Background:** Application of the capsaicin 8% patch is commonly associated with treatment-related burning pain, which may necessitate rescue analgesia, compromise completion of the standardized 60 min application, and reduce acceptance of repeat treatment. Although capsaicin is an established therapy for painful diabetic peripheral neuropathy (PDPN), evidence supporting strategies to improve procedural tolerability remains limited. This study evaluated whether ultrasound-guided bilateral ankle nerve block (UGBANB) was associated with better procedural outcomes than topical eutectic mixture of lidocaine/prilocaine (EMLA) pretreatment or no pretreatment. **Methods:** This retrospective comparative cohort study included 90 adults with neurologist-confirmed PDPN who underwent capsaicin 8% patch therapy. Patients received no pretreatment (n = 30), topical EMLA pretreatment (n = 30), or UGBANB (n = 30). The primary outcome was cumulative procedural pain burden, quantified as the area under the Numeric Pain Rating Scale–time curve (AUC-NPRS) during the standardized 60 min application. Secondary outcomes included rescue tramadol requirements, treatment completion, early postprocedural pain intensity, procedural tolerability, willingness to undergo repeat treatment, and safety. **Results:** Cumulative procedural pain burden differed significantly among the three pretreatment strategies (one-way ANOVA: F = 289.8, *p* < 0.001; η^2^ = 0.87). Mean AUC-NPRS was 481.5 ± 39.2 NPRS·min with no pretreatment, 429.2 ± 36.0 with topical EMLA, and 256.3 ± 38.4 with UGBANB, corresponding to approximately 47% and 40% lower cumulative pain burden with UGBANB than with no pretreatment and topical EMLA, respectively. No patient receiving UGBANB required intravenous rescue tramadol, compared with 60.0% receiving EMLA and 100.0% receiving no pretreatment (*p* < 0.001). Treatment completion was 100% with UGBANB, compared with 83.3% with EMLA and 73.3% with no pretreatment. UGBANB was also associated with lower early postprocedural pain, greater procedural tolerability, and greater willingness to undergo repeat treatment. No serious treatment- or block-related adverse events were observed during the available follow-up. **Conclusions:** UGBANB was associated with better procedural tolerability and treatment delivery during capsaicin 8% patch therapy than topical EMLA or no pretreatment in patients with PDPN. These findings relate specifically to procedural optimization and should not be interpreted as evidence that UGBANB enhances the intrinsic or long-term analgesic efficacy of capsaicin. Prospective multicenter randomized controlled trials are needed to confirm these associations and determine whether improved procedural tolerability translates into better long-term treatment adherence, clinical outcomes, health-related quality of life, safety, and cost-effectiveness.

## 1. Introduction

Painful diabetic peripheral neuropathy (PDPN) is one of the most common and disabling complications of diabetes mellitus, affecting approximately one-third of individuals with longstanding disease and substantially impairing quality of life. It is characterized by spontaneous burning pain, paresthesia, hyperalgesia, and allodynia, symptoms that interfere with sleep, mobility, daily functioning, and psychological well-being. While guideline-recommended oral pharmacological therapies are the cornerstone of management, many patients experience inadequate pain relief, treatment-limiting adverse effects, or contraindications to long-term systemic therapy [1,2,3,4,5]. Localized therapies that target peripheral nociceptive fibers while minimizing systemic drug exposure have therefore become increasingly important in contemporary management, particularly as PDPN predominantly affects the distal feet [6,7,8,9,10].

The capsaicin 8% dermal patch is an established therapy for localized peripheral neuropathic pain, including PDPN [6,11,12,13,14,15,16]. Capsaicin selectively activates transient receptor potential vanilloid 1 (TRPV1) receptors, causing reversible defunctionalization of nociceptive nerve endings and producing sustained analgesia after a single supervised application [11,17,18]. International guidelines recommend high-concentration capsaicin as a treatment option for patients with PDPN, particularly when systemic therapies provide inadequate relief, are poorly tolerated, or are contraindicated [6,7,8,9,10]. Randomized controlled trials, long-term studies, and real-world investigations have demonstrated its efficacy and safety across clinical and routine-practice settings [12,15,16,19,20]. Unlike oral therapies, capsaicin is administered as a supervised outpatient procedure requiring standardized application, monitoring, and post-treatment assessment [16,20,21,22]. Because its analgesic benefit may diminish over time, repeated applications may be required [14,15,16]. Consequently, successful treatment depends not only on analgesic efficacy but also on patients’ ability to complete the recommended application and their willingness to undergo repeat treatment when clinically indicated [15,16,21].

Despite its established analgesic efficacy, capsaicin 8% patch application is frequently associated with intense, transient burning pain during the standard treatment period. Although this discomfort is generally self-limiting, it may necessitate rescue analgesia, contribute to premature patch removal, and discourage patients from undergoing subsequent treatment sessions [16,21,22]. Consequently, improving procedural tolerability and minimizing rescue opioid use have become important objectives in contemporary multimodal pain management [23,24]. Because successful treatment depends on patients’ ability to complete and accept the full application protocol, procedural outcomes such as treatment completion, acceptability, and willingness to undergo repeat therapy represent clinically relevant measures alongside analgesic efficacy [25,26,27].

Topical pretreatment with a eutectic mixture of lidocaine and prilocaine (EMLA) is commonly used to reduce application-related discomfort during capsaicin 8% patch therapy [16,21,22]. Nevertheless, clinically significant burning pain may persist, and rescue analgesia may still be required to facilitate completion of the standard 60-min application [16,21,22,28]. Regional anesthesia represents an alternative strategy to temporarily interrupt peripheral nociceptive transmission before capsaicin exposure. In patients with PDPN, ultrasound-guided bilateral ankle nerve block (UGBANB) provides targeted sensory blockade of the distal foot through blockade of the terminal peripheral nerves supplying the treated area [29,30,31,32,33,34]. Ultrasound guidance facilitates direct visualization of neural and vascular structures and local anesthetic spread, improving block accuracy and procedural success while potentially reducing technical complications [30,31,33,35]. Although peripheral nerve blocks are routinely used for foot and ankle procedures [29,30,31,32], their application as a procedural analgesic strategy for capsaicin 8% patch therapy has not been widely investigated. Recent evidence from a retrospective cohort study suggests that ultrasound-guided thoracic paravertebral block may improve procedural tolerability during capsaicin 8% patch treatment for thoracic postherpetic neuralgia [36]; however, whether comparable procedural benefits can be achieved with distal lower-extremity nerve blockade in PDPN remains uncertain.

Accordingly, this retrospective comparative cohort study evaluated whether UGBANB was associated with improved procedural tolerability during capsaicin 8% patch therapy in adults with neurologist-confirmed PDPN, compared with topical EMLA pretreatment or no pretreatment. We compared cumulative procedural pain burden, rescue analgesic requirements, treatment completion, early postprocedural pain intensity, procedural tolerability, willingness to undergo repeat treatment, and safety. We hypothesized that UGBANB would be associated with improved procedural tolerability and more favorable patient-reported and safety outcomes than topical EMLA pretreatment or no pretreatment.

## 2. Materials and Methods

### 2.1. Study Design

This single-center retrospective comparative cohort study reviewed the electronic medical records of consecutive adults who underwent capsaicin 8% patch therapy for PDPN at the Pain Medicine Clinic, University of Tabuk, Saudi Arabia, between January 2023 and December 2025. The study was conducted in accordance with the Declaration of Helsinki and approved by the Research Ethics Committee of the University of Tabuk, Saudi Arabia (Approval No. UT-637-396-2025). Standard informed consent for clinical treatment was obtained as part of routine care. No additional informed consent was required for this retrospective analysis of anonymized clinical data in accordance with Research Ethics Committee approval.

The study is reported in accordance with the Strengthening the Reporting of Observational Studies in Epidemiology (STROBE) guidelines for cohort studies. Clinical, procedural, and follow-up data were extracted from electronic medical records, using a predefined data abstraction framework and standardized variable definitions to ensure consistent data collection across all pretreatment groups.

### 2.2. Clinical Setting and Referral Pathway

The study was conducted at the Pain Medicine Clinic, University of Tabuk, Saudi Arabia, a tertiary referral center providing multidisciplinary management for chronic pain disorders. Adults with neurologist-confirmed PDPN were referred from the Departments of Neurology, Endocrinology/Diabetology, Primary Care, Orthopedic Surgery, Vascular Surgery, and other specialties. Referrals followed inadequate response, intolerance, or contraindication to guideline-recommended pharmacological therapy, or when capsaicin 8% patch therapy was considered appropriate for persistent localized neuropathic pain despite optimized medical management.

The diagnosis of PDPN was established by the referring consultant neurologist according to the diagnostic criteria described in Section 2.4. After referral, all patients underwent a standardized evaluation by a fellowship-trained consultant in pain medicine. This confirmed eligibility for capsaicin 8% patch therapy, excluded contraindications, and documented demographic characteristics, diabetes history, neuropathic pain features, current medications, glycemic status, and relevant comorbidities.

During the study period, 112 consecutive patients were screened. After excluding patients who did not meet the predefined inclusion criteria or had incomplete procedural or follow-up documentation, 90 participants were included in the final analysis. As part of routine clinical practice, patients received one of three pretreatment strategies before capsaicin application: no pretreatment, topical EMLA, or UGBANB. Treatment selection reflected routine clinical decision-making rather than a predefined study allocation.

**Figure 1:**
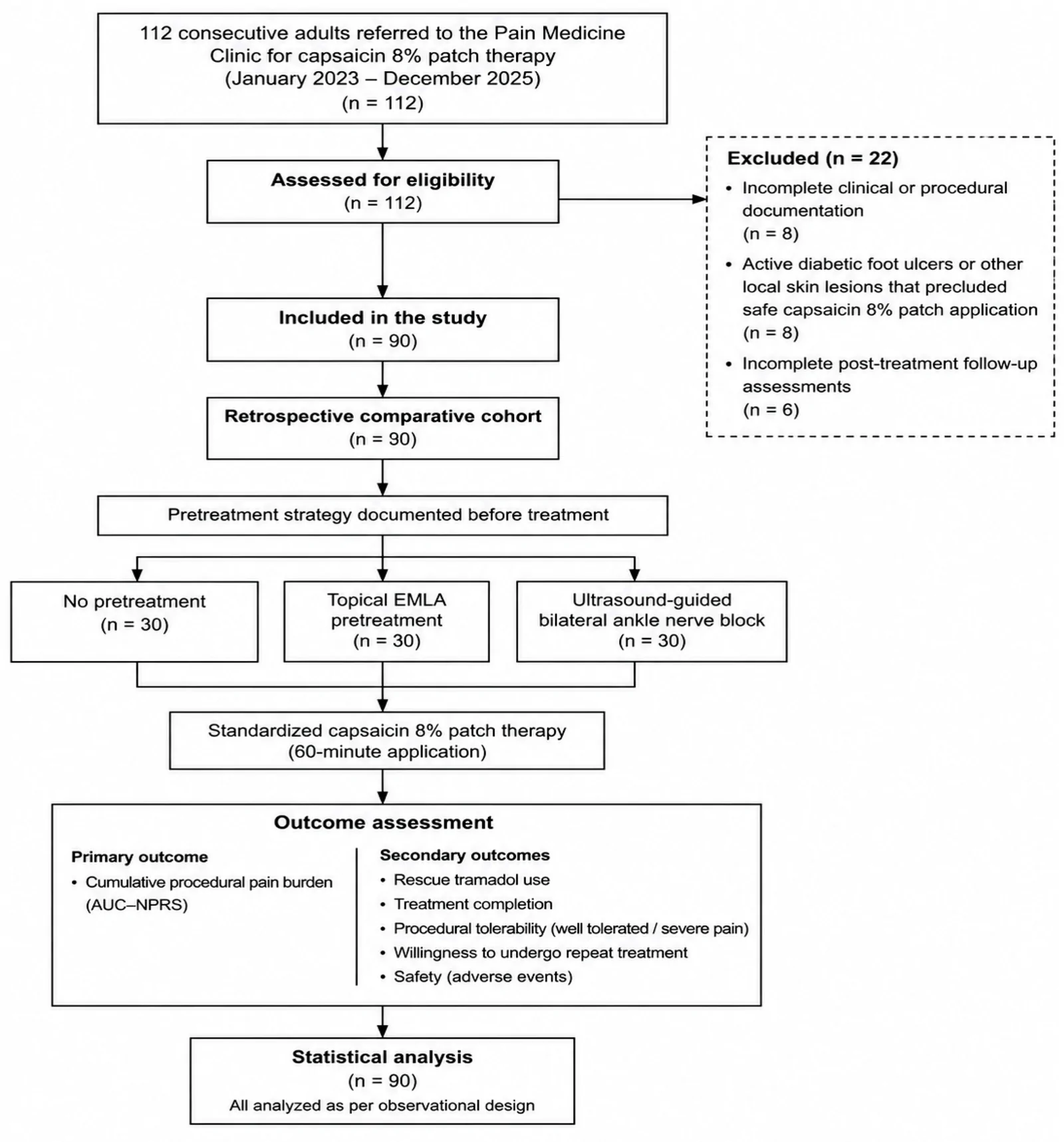
Participant selection, pretreatment classification, treatment pathway, and outcome assessment in this retrospective comparative cohort study.

All pretreatment strategies were available to patients throughout the study period and were selected according to clinician judgment, patient preference, and operator availability. Referral pathways, eligibility criteria, treatment protocols, pain assessment procedures, rescue analgesia thresholds, and follow-up schedules remained unchanged throughout the study. This ensured a standardized institutional clinical pathway across all pretreatment groups. The overall study pathway is summarized in Figure 1.

### 2.3. Patient Selection

Consecutive adults (≥18 years) who underwent capsaicin 8% patch therapy for PDPN during the study period were screened for inclusion. Patients were eligible after inadequate response, intolerance, or contraindication to guideline-recommended pharmacological therapies [2,3,4,5,6,7,8,9,10], provided complete procedural and follow-up data were available.

Inclusion criteria were as follows: (1) age ≥18 years; (2) type 2 diabetes mellitus for ≥2 years; (3) neurologist-confirmed PDPN affecting one or both feet; (4) neuropathic pain duration ≥6 months; (5) baseline Numeric Pain Rating Scale (NPRS) score ≥4; (6) inadequate response, intolerance, or contraindication to at least one guideline-recommended first-line pharmacological therapy [2,3,4,5,6,7,8,9,10]; and (7) intact skin over the intended capsaicin application site.

Persistent neuropathic pain despite treatment with pregabalin (≥150 mg/day) or gabapentin (≥900 mg/day), with or without amitriptyline (≥10 mg/day), was considered an inadequate response. Documented intolerance to these medications was considered treatment failure. Baseline neuropathic pain medications were continued throughout the study and were not modified because of capsaicin therapy.

Exclusion criteria included active local skin infection; diabetic foot ulceration at the treatment site; clinically significant peripheral arterial disease; hypersensitivity to capsaicin, local anesthetics, or patch components; previous lower-extremity peripheral nerve block within three months; pregnancy or breastfeeding; cognitive impairment preventing reliable pain assessment; and incomplete medical records that precluded outcome assessment.

### 2.4. Diagnosis of Painful Diabetic Peripheral Neuropathy

PDPN diagnosis was established by the referring consultant neurologist according to contemporary international recommendations and was independently confirmed by a fellowship-trained consultant in Pain Medicine before capsaicin 8% patch therapy. Diagnosis required a compatible history of type 2 diabetes mellitus, chronic symmetrical distal neuropathic pain affecting one or both feet, characteristic neuropathic symptoms (burning pain, tingling, paresthesia, hyperalgesia, and allodynia), and sensory abnormalities on neurological examination consistent with distal symmetrical polyneuropathy [4,37,38].

During clinical assessment, the Pain Medicine consultant considered the neurologist’s documentation alongside diabetes duration, neuropathic pain history, previous and current medications, glycemic status, and relevant laboratory or imaging findings to exclude alternative causes of peripheral neuropathy or lower-extremity pain. Nerve conduction studies and electromyography were considered when clinically indicated and available but were not required for study eligibility, consistent with contemporary recommendations emphasizing clinical assessment in typical distal symmetrical diabetic polyneuropathy [1,5,12].

Although validated neuropathic pain screening instruments, including the Douleur Neuropathique 4 (DN4) questionnaire and painDETECT, were available, they were not routinely used. Diagnosis therefore relied on specialist clinical assessment supported by contemporaneous neurological documentation. Independent confirmation by the referring neurologist and a fellowship-trained Pain Medicine consultant strengthened diagnostic consistency and ensured appropriate patient selection for capsaicin 8% patch therapy.

### 2.5. Pretreatment Classification

Participants were categorized according to the pretreatment received before capsaicin 8% patch therapy: no pretreatment, topical pretreatment with EMLA, or UGBANB.

Pretreatment was selected as part of routine clinical practice, rather than through randomization. Selection was based on the clinical judgment of the treating fellowship-trained consultant in pain medicine, patient preference when applicable, and operator availability. All three strategies remained available to patients throughout the study period.

All participants subsequently underwent an identical capsaicin treatment protocol, including standardized pain assessments, rescue analgesia, postprocedural care, and follow-up.

As treatment allocation was non-randomized, selection bias, confounding by indication, and residual confounding cannot be excluded. Nevertheless, standardized eligibility criteria, treatment procedures, outcome measures, and follow-up reduced methodological variability. Therefore, between-group comparisons should be interpreted as observational associations rather than causal effects.

### 2.6. Ultrasound-Guided Bilateral Ankle Nerve Block

Patients assigned to the regional anesthesia group underwent UGBANB approximately 30 min before capsaicin 8% patch therapy. All procedures were performed by fellowship-trained Pain Medicine consultants experienced in peripheral nerve blockade, using a standardized institutional protocol consistent with contemporary descriptions of ultrasound-guided ankle block techniques [32,33,34,35,36].

After skin antisepsis, a high-frequency linear ultrasound transducer (10–15 MHz; SonoSite Edge II, Fujifilm SonoSite, Bothell, WA, USA) was used to identify the posterior tibial, deep peroneal, superficial peroneal, sural, and saphenous nerves. A 22-gauge, 50 mm insulated block needle was advanced using an in-plane approach under real-time ultrasound guidance without peripheral nerve stimulation. Each foot received 10 mL of 0.25% bupivacaine (2 mL around each target nerve), administered incrementally after repeated negative aspiration. Adequate perineural spread was verified sonographically, with needle repositioning performed when necessary to optimize injectate distribution.

Patients remained under standard clinical monitoring until block onset was verified by reduced or absent sensation to pinprick, cold, and light touch within the distribution of the targeted nerves. Capsaicin treatment commenced only after satisfactory blockade had been achieved, typically 30 min after injection.

After the standardized 60 min capsaicin application, patients underwent routine postprocedural assessment before discharge. Discharge criteria included stable vital signs, adequate pain control, and safe ambulation. Standardized verbal and written instructions were provided regarding temporary sensory impairment, protection of the anesthetized feet, avoidance of unassisted ambulation until return of sensation, and avoidance of driving or operating hazardous machinery until complete block resolution. These precautions were intended to minimize inadvertent injury during transient sensory impairment, particularly in patients with diabetic peripheral neuropathy who may already have impaired protective sensation and increased susceptibility to balance impairment and falls [30,39,40,41].

### 2.7. Capsaicin 8% Patch Procedure

Capsaicin 8% dermal patches (Qutenza, Averitas Pharma, Inc., Morristown, NJ, USA) were applied according to the manufacturer’s prescribing information and institutional protocol, consistent with contemporary recommendations for high-concentration capsaicin therapy [16,21,22]. The procedure was identical for all participants, irrespective of pretreatment group.

After the assigned pretreatment and confirmation of adequate sensory blockade when applicable, capsaicin was applied by a trained general practitioner under the supervision of a fellowship-trained consultant in Pain Medicine. Painful areas on one or both feet were identified according to the patient’s reported pain distribution and clinical examination. The treatment area was outlined with a skin marker, and one or more patches were trimmed to conform to the affected anatomical regions while minimizing exposure of unaffected skin. The number of patches used was determined by the size and distribution of the painful areas. Application time was standardized at 60 min.

Following skin cleansing and drying, the patches were applied and secured according to the manufacturer’s instructions. Nitrile gloves were worn throughout patch preparation, application, and removal. No additional topical anesthetics, cooling measures, or adjunctive analgesic interventions were used beyond the assigned pretreatment and the standardized rescue analgesia protocol described in Section 2.10.

Continuous nursing observation was maintained throughout the application period. Pain intensity was assessed according to the predefined assessment schedule, and rescue analgesia was administered according to the standardized institutional protocol described in Section 2.10. Verbal reassurance was provided as part of routine clinical care to facilitate treatment completion.

After patch removal, the treated skin was cleansed using the proprietary cleansing gel supplied with the treatment kit. Patients received standardized verbal and written instructions regarding expected transient application-site reactions, appropriate skin care, the avoidance of excessive heat exposure, and the use of rescue analgesia when required. All procedural observations were documented contemporaneously in the electronic medical record.

### 2.8. Outcome Measures and Assessment

Outcome data were retrospectively extracted from electronic medical records using a predefined data abstraction form developed before data collection. The principal investigator performed all data extraction using prespecified variable definitions and uniform eligibility criteria. Because only patients with complete procedural and follow-up documentation were included, no imputation of missing data was required.

Pain intensity was prospectively recorded during routine clinical care by experienced nursing staff using the validated 11-point NPRS (0 = no pain, 10 = worst imaginable pain). Assessments were performed at baseline, then every 10 min during the 60 min capsaicin application (10, 20, 30, 40, 50, and 60 min), and at 8, 24, and 48 h after treatment.

Following the institutional rescue analgesia protocol, intravenous tramadol was administered when the NPRS score was ≥7 despite supportive measures, or earlier if pain was considered intolerable by the treating pain physician. The same criteria were applied across all study groups.

Because pretreatment strategies were visually distinguishable, blinding of nursing staff was not feasible. However, they had no involvement in the study design, data extraction, statistical analysis, or interpretation. Pain scores, rescue analgesia use, treatment completion, patient-reported outcomes, and safety events were prospectively recorded according to institutional clinical practice before study initiation, reducing the likelihood of differential outcome ascertainment.

Information on rescue analgesia, treatment completion, patient-reported outcomes, and adverse events was obtained from the contemporaneous clinical records using prespecified definitions. Safety follow-up extended through the scheduled 48 h assessment and subsequent outpatient review, where available, and included local treatment reactions, block-related complications, delayed neurological symptoms, and other clinically significant adverse events.

Longitudinal pain measurements were analyzed using repeated-measures methods. Categorical outcomes were analyzed using the statistical approaches outlined below. Detailed repeated-measures results, including Greenhouse–Geisser corrections, effect sizes, and Bonferroni-adjusted pairwise comparisons, are provided in Supplementary Table S1.

### 2.9. Study Outcomes

#### 2.9.1. Primary Outcome

The primary outcome was cumulative procedural pain burden during the standardized 60 min capsaicin 8% patch application, quantified as the area under the Numeric Pain Rating Scale–time curve (AUC-NPRS). AUC-NPRS was calculated using the trapezoidal rule and integrated serial pain intensity measurements over time to provide a cumulative measure of procedural pain burden [42,43,44].

#### 2.9.2. Secondary Clinical Outcomes

Secondary clinical outcomes included intravenous rescue tramadol administration during the application period, cumulative oral tramadol consumption during the first 48 h after treatment, completion of the planned 60 min application, and postprocedural pain intensity at 8, 24, and 48 h.

#### 2.9.3. Patient-Reported Outcomes

Patient-reported outcomes included procedural tolerability and willingness to undergo repeat capsaicin treatment. Procedural tolerability reflected the participant’s overall treatment experience and was categorized as well tolerated, moderate discomfort, or severe burning pain according to predefined study definitions applied to contemporaneous clinical documentation. Willingness to undergo repeat treatment was recorded as a documented yes/no response during routine follow-up. These outcomes were selected to complement conventional pain measures by capturing treatment acceptability and patient experience, which are relevant to treatment engagement and real-world implementation [25,26,27].

#### 2.9.4. Safety Outcomes

Safety outcomes included local application-site reactions (erythema, blistering, and skin irritation), block-related complications (vascular puncture, prolonged sensory blockade, clinically significant motor weakness, local anesthetic systemic toxicity [LAST], persistent sensory deficits, and infection), treatment-related hospitalization, postprocedural falls, delayed discharge, and other treatment-related adverse events documented during the observation period and routine follow-up.

### 2.10. Statistical Analysis

Because of the retrospective exploratory cohort design, no formal a priori sample size calculation was performed.

Continuous data are presented as the mean ± standard deviation (SD) or median (interquartile range [IQR]) according to distribution, while categorical data are presented as a number (%). Normality and homogeneity of variance were assessed using the Shapiro–Wilk and Levene’s tests, respectively. Baseline characteristics were compared using one-way ANOVA for continuous variables, and the chi-squared or Fisher’s exact test for categorical variables.

The primary endpoint, cumulative procedural pain burden, was quantified as the AUC-NPRS using the trapezoidal rule. For participants who discontinued treatment before 60 min, the AUC-NPRS was calculated from observed pain measurements only, without extrapolation or imputation. Between-group differences were evaluated using one-way ANOVA with Tukey’s honestly significant difference (HSD) post hoc test. Effect sizes are reported as eta squared (η^2^) and Cohen’s *d*.

Longitudinal pain scores obtained during treatment and at 8, 24, and 48 h were analyzed using repeated-measures ANOVA. Sphericity was assessed with Mauchly’s test, and the Greenhouse–Geisser correction was applied as required. Bonferroni-adjusted pairwise comparisons followed significant overall effects. Effect sizes are reported as partial eta squared (partial η^2^). Detailed results are provided in Supplementary Table S1.

Secondary efficacy, patient-reported, and safety endpoints were compared using one-way ANOVA, the chi-squared test, or Fisher’s exact test, as appropriate. Categorical results are presented as risk ratios (RRs) with 95% confidence intervals (CIs), while selected continuous variables are reported as mean differences (MDs) with 95% CIs. In cases of zero-event cells, continuity-corrected estimates were used.

Missing data were minimal and handled by complete-case analysis. Owing to the retrospective design and modest sample size, multivariable regression, propensity score methods, inverse probability weighting, and sensitivity analyses were not undertaken because of the risk of unstable estimates and model overfitting. Other than Bonferroni adjustment in the repeated-measures analyses, no correction for multiple testing was applied to secondary endpoints, which should therefore be interpreted as exploratory. Accordingly, between-group comparisons indicate associations rather than causal effects.

All statistical tests were two-sided, and *p* < 0.05 was considered statistically significant. Statistical analyses were performed using IBM SPSS Statistics version 27.0 (IBM Corp., Armonk, NY, USA).

## 3. Results

### 3.1. Study Population

During the study period, the records of 112 consecutive adults referred for capsaicin 8% patch therapy were screened (Figure 1). Twenty-two were excluded before group allocation: eight due to incomplete clinical or procedural documentation; eight because of active diabetic foot ulcers or other local skin lesions precluding treatment; and six due to incomplete follow-up assessments. The remaining 90 patients were included in the final analysis.

The cohort comprised 30 patients in each of the no pretreatment, EMLA, and UGBANB groups. Complete outcome data were available for all patients, and no imputation was required. Baseline demographic and clinical characteristics are summarized in Table 1.

### 3.2. Baseline Demographic and Clinical Characteristics

Participants’ baseline demographic and clinical characteristics are presented in Table 1. The three study groups were comparable across all measured demographic and clinical variables; no statistically significant between-group differences were observed in age, sex, body mass index, diabetes duration, PDPN duration, glycated hemoglobin (HbA1c), NPRS score, smoking status, bilateral foot involvement, comorbidities, or concomitant neuropathic pain medications (all *p* > 0.05). All participants had type 2 diabetes mellitus, and none had previously received capsaicin 8% patch therapy.

### 3.3. Primary Outcome: Cumulative Procedural Pain Burden

**Figure 2:**
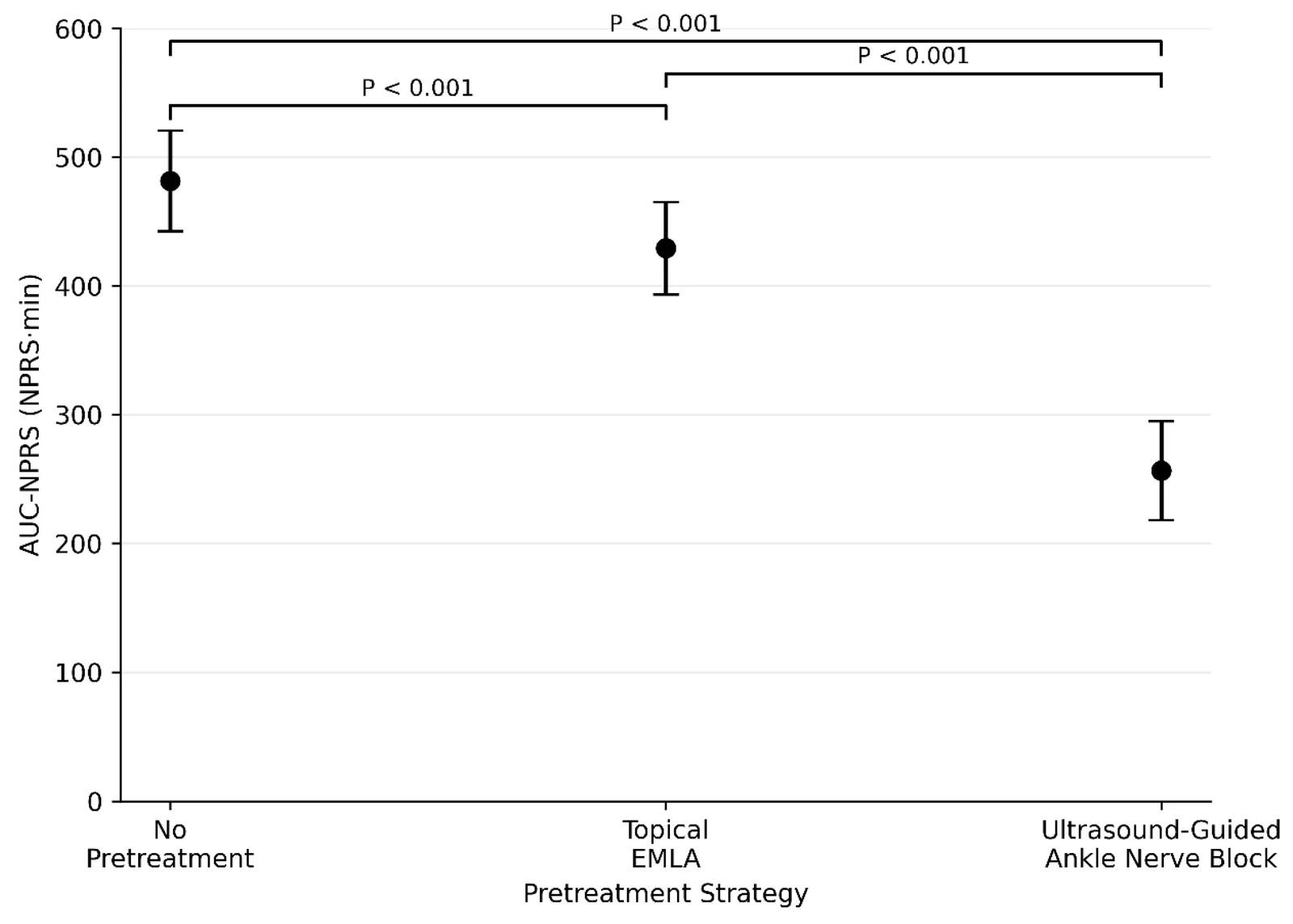
Cumulative Procedural Pain Burden According to Pretreatment Strategy. Mean cumulative procedural pain burden during the standardized 60 min capsaicin 8% patch application, expressed as the area under the Numeric Pain Rating Scale–time curve (AUC-NPRS), according to pretreatment strategy. Error bars represent standard deviation. Overall between-group differences were significant (*p* < 0.001), with pairwise comparisons showing progressively lower AUC-NPRS from no pretreatment to topical EMLA pretreatment and ultrasound-guided bilateral ankle nerve block. Abbreviations: AUC-NPRS, area under the Numeric Pain Rating Scale–time curve; EMLA, eutectic mixture of lidocaine and prilocaine.

The cumulative procedural pain burden during the 60 min capsaicin 8% patch application, as quantified by AUC-NPRS, differed significantly among the three groups (Table 2, Figure 2). Mean AUC-NPRS was highest without pretreatment (481.5 ± 39.2 NPRS·min), intermediate with topical EMLA (429.2 ± 36.0 NPRS·min), and lowest with UGBANB (256.3 ± 38.4 NPRS·min).

Overall differences were highly significant (one-way ANOVA: F = 289.8, *p* < 0.001; η^2^ = 0.87). Post hoc analysis showed lower AUC-NPRS with topical EMLA than with no pretreatment, whereas UGBANB was associated with the lowest values and differed significantly from both comparator groups (all adjusted *p* < 0.001). Corresponding effect sizes (Cohen’s d) were 1.39 for EMLA versus no pretreatment and 4.64–5.80 for comparisons involving UGBANB.

### 3.4. Procedural Pain Trajectory During Capsaicin Application

**Figure 3:**
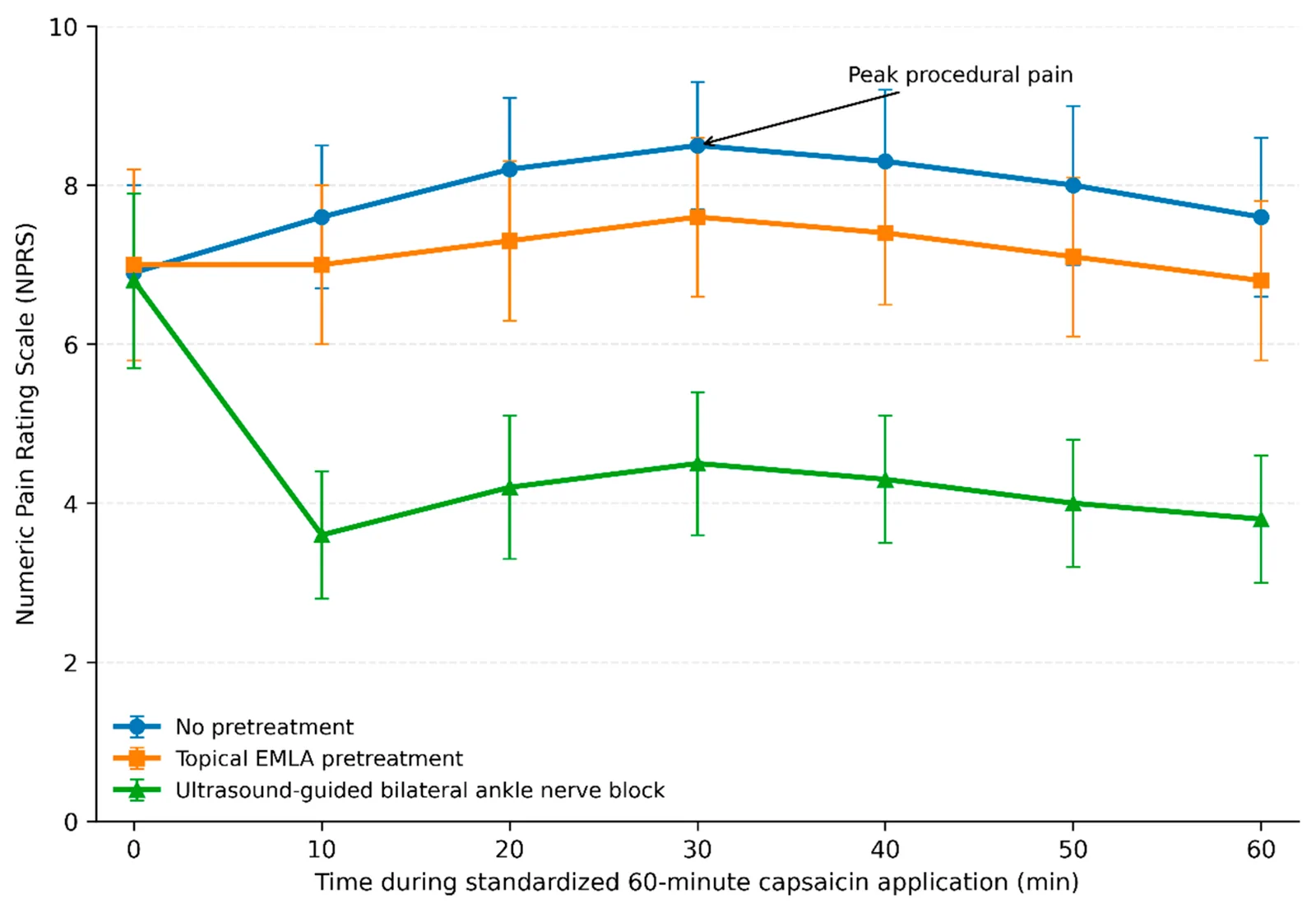
Procedural Pain Intensity During the Standardized 60-min Capsaicin 8% Patch Application According to Pretreatment Strategy. Mean Numeric Pain Rating Scale (NPRS) scores during the standardized 60 min capsaicin 8% patch application according to pretreatment strategy. Error bars represent standard deviation (SD). Pain intensity increased during the early phase of treatment, reached a peak at approximately 30 min, and subsequently declined toward the end of the application period. Throughout the procedure, pain scores remained lowest in the ultrasound-guided bilateral ankle nerve block group, intermediate following topical EMLA pretreatment, and highest in the no pretreatment group. Statistical comparisons over time are summarized in the Results Section, with detailed repeated-measures ANOVA and Bonferroni-adjusted pairwise comparisons provided in Supplementary Table S1. Abbreviations (Footnote): EMLA, eutectic mixture of lidocaine and prilocaine; NPRS, Numeric Pain Rating Scale; SD, standard deviation.

Serial NPRS measurements demonstrated distinct pain patterns across the three groups (Figure 3, Table 3). In all groups, scores increased during the early phase of treatment, peaked at approximately 30 min, and gradually declined thereafter. At each post-baseline assessment, mean NPRS scores were lowest with UGBANB, intermediate with topical EMLA, and highest without pretreatment. Overall between-group differences at each post-baseline assessment were significant (all *p* < 0.001; Table 3).

Repeated-measures ANOVA demonstrated a significant effect of time on procedural pain (F(6, 534) = 17.36, *p* < 0.001; partial η^2^ = 0.163). Bonferroni-adjusted pairwise comparisons identified significant differences between several procedural time points, as detailed in Supplementary Table S1.

### 3.5. Rescue Analgesia Requirements

Rescue analgesic requirements differed significantly among the three groups during capsaicin 8% patch application and throughout the subsequent 48 h follow-up (Table 4). Intravenous rescue tramadol was required by all patients without pretreatment, 60.0% of those receiving topical EMLA, and none receiving UGBANB (*p* < 0.001). Among patients requiring rescue analgesia, cumulative intravenous tramadol consumption was significantly lower in those receiving EMLA than without pretreatment.

The same pattern was observed during follow-up. Oral rescue tramadol was required by all patients without pretreatment, compared with 60.0% and 20.0% of those in the EMLA and UGBANB groups, respectively (*p* < 0.001). Cumulative oral tramadol consumption also showed a stepwise reduction across the three groups, with the lowest requirements observed in the UGBANB group. RRs, MDs, and their corresponding 95% CIs are summarized in Table 4.

### 3.6. Postprocedural Pain Intensity

**Figure 4:**
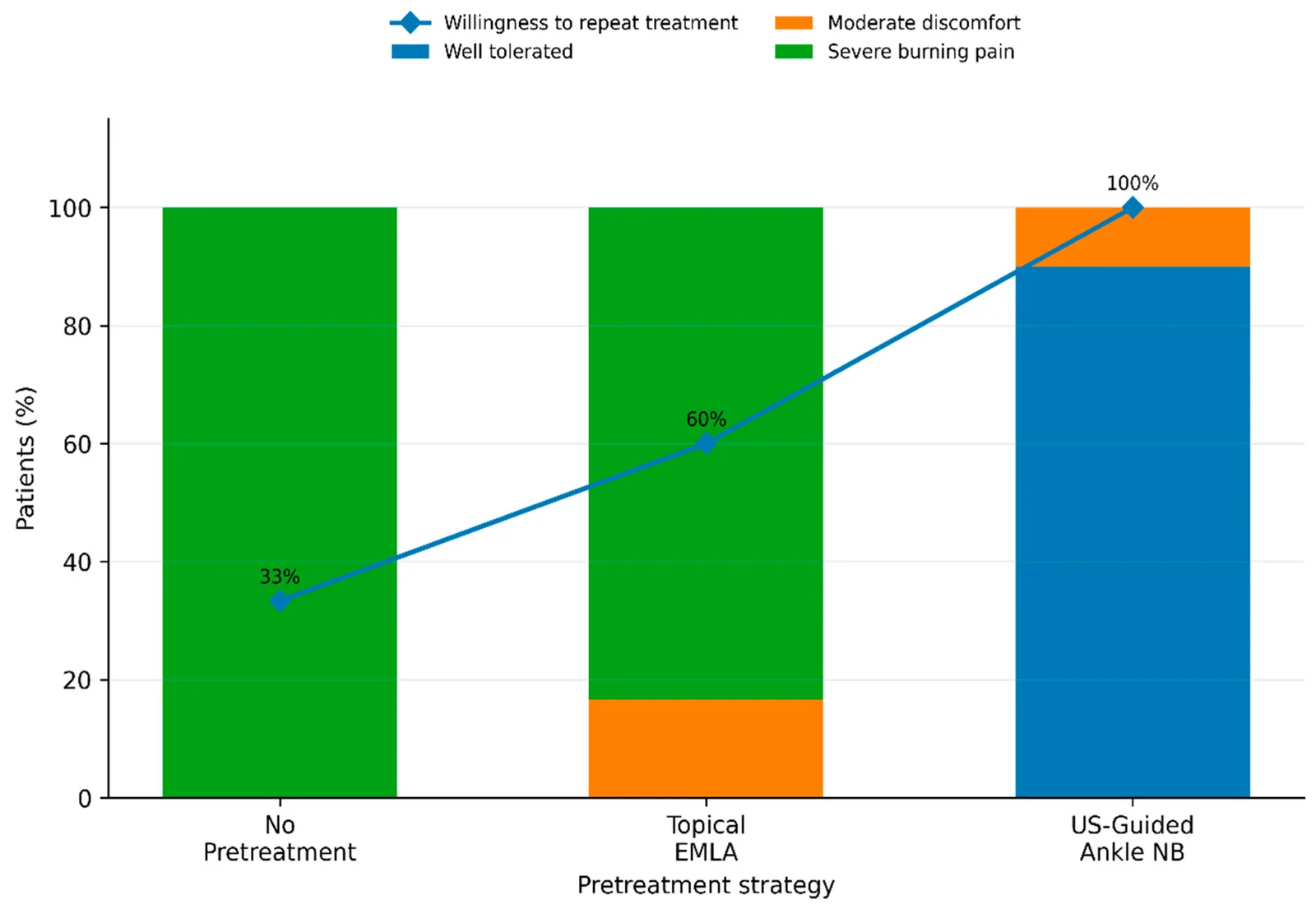
Patient-Reported Procedural Tolerability and Willingness to Repeat Capsaicin 8% Patch Therapy. The distribution of patient-reported procedural tolerability and willingness to undergo repeat capsaicin 8% patch therapy according to pretreatment strategy. Stacked bars show the proportions of patients reporting the procedure as well tolerated, associated with moderate discomfort, or associated with severe procedural burning pain. Diamond markers indicate the percentage of patients willing to undergo repeat capsaicin 8% patch treatment. Ultrasound-guided bilateral ankle nerve block was associated with the highest proportion of well-tolerated procedures and the greatest willingness to repeat treatment, whereas severe procedural burning pain predominated in the no pretreatment and topical EMLA groups.

Postprocedural pain declined progressively during the 48 h follow-up (Table 5, Figure 4). Mean NPRS scores decreased from 4.58 ± 1.34 at 8 h to 3.74 ± 1.11 at 24 h and 3.17 ± 1.00 at 48 h.

Repeated-measures ANOVA demonstrated a significant effect of time (F(2,178) = 231.36, *p* < 0.001; partial η^2^ = 0.722), consistent with a progressive reduction in pain during follow-up. Bonferroni-adjusted pairwise comparisons confirmed significant decreases between 8 and 24 h, 24 and 48 h, and 8 and 48 h (all adjusted *p* < 0.001). Detailed repeated-measures results are provided in Supplementary Table S1.

### 3.7. Treatment Completion

Treatment completion differed significantly among the three groups (Table 6). All patients receiving UGBANB completed the planned 60 min capsaicin 8% patch application, compared with 83.3% of those in the topical EMLA group and 73.3% without pretreatment (*p* = 0.018).

Premature discontinuation occurred in 26.7% of patients without pretreatment and 16.7% receiving EMLA, whereas no patient in the UGBANB group discontinued the application. Among those who discontinued, the median application duration was 36 min (IQR, 33–39) without pretreatment and 39 min (IQR, 35–42) with EMLA.

### 3.8. Patient-Reported Outcomes

Patient-reported outcomes differed significantly among the three groups (Table 7). Procedural tolerability differed significantly among the groups, with progressively more favorable ratings from no pretreatment to topical EMLA and UGBANB (*p* < 0.001). In the UGBANB group, 90.0% of patients rated the procedure as well tolerated and 10.0% reported moderate discomfort, with no reports of severe procedural burning pain. In contrast, severe burning pain was reported by 83.3% of patients receiving EMLA and by all patients without pretreatment.

Willingness to undergo repeat capsaicin 8% patch therapy also differed significantly (*p* < 0.001). Repeat treatment was considered acceptable by 33.3%, 60.0%, and 100.0% of patients in the no pretreatment, EMLA, and UGBANB groups, respectively. Compared with no pretreatment, acceptance was 1.80-fold higher with EMLA (RR = 1.80, 95% CI 1.01–3.22) and 3.00-fold higher with UGBANB (RR = 3.00, 95% CI 1.76–5.12) (Table 7).

### 3.9. Safety Outcomes

Safety outcomes are summarized in Table 8. Transient localized erythema at the application site occurred in all patients (90/90, 100%) and resolved spontaneously without additional intervention.

No serious adverse events occurred during the study period. No blistering, skin ulceration, secondary infection, persistent sensory deficits, delayed neurological complications, systemic toxicity, or treatment-related hospitalization were recorded. In the UGBANB group, no block-related complications, including LAST or vascular puncture, were observed.

## 4. Discussion

### 4.1. Principal Findings

This retrospective comparative cohort study evaluated three pretreatment strategies for optimizing the delivery of capsaicin 8% patch therapy in patients with neurologist-confirmed PDPN who remained symptomatic despite guideline-recommended pharmacological management or were unable to continue first-line therapy because of inadequate response, intolerance, or contraindications. Whereas previous studies have primarily examined the analgesic efficacy, safety, and longer-term effectiveness of high-concentration capsaicin, the present study addressed the comparatively underexplored challenge of improving procedural tolerability during the standardized 60 min application period [7,11,12,13,14,15,16,20,28,45,46].

UGBANB was consistently associated with better procedural and early postprocedural outcomes than topical EMLA or no pretreatment. The primary endpoint—cumulative procedural pain burden measured by AUC-NPRS—differed significantly among the three groups (F = 289.8, *p* < 0.001; η^2^ = 0.87). Mean AUC-NPRS was approximately 47% lower with UGBANB than with no pretreatment and 40% lower than with topical EMLA. The pattern of association was consistent across secondary outcomes. Patients receiving UGBANB required no intravenous rescue tramadol during application, had lower oral tramadol requirements during the subsequent 48 h, achieved a higher treatment completion rate, and reported lower early postprocedural pain, greater procedural tolerability, and greater willingness to undergo repeat capsaicin therapy. These findings were observed without an apparent increase in short-term block-related complications or other serious adverse events.

Collectively, these findings suggest that optimizing procedural analgesia, rather than modifying the intrinsic pharmacological action of capsaicin, may improve the conditions under which capsaicin 8% patch therapy is delivered and accepted by patients. Although the retrospective observational design precludes causal inference, the consistency of the observed associations across procedural, patient-reported, and short-term safety outcomes provides a rationale for prospective evaluation of regional anesthesia-assisted capsaicin treatment pathways.

### 4.2. Procedural Optimization of Capsaicin Therapy

The present findings highlight the distinction between the established analgesic efficacy of capsaicin 8% patch therapy and the tolerability of its administration. Randomized trials, long-term studies, and contemporary clinical recommendations support high-concentration capsaicin as a treatment option for painful peripheral neuropathic conditions, including PDPN [6,7,8,9,10,12,13,14,15,16,20,28] however, treatment-related burning pain during the standardized application remains a recognized barrier to procedural tolerability and treatment delivery [16,19,21,22,24,27,28,46]. Accordingly, improving the application experience without altering the pharmacological action of capsaicin may facilitate its implementation in routine clinical practice. In this cohort, pretreatment with UGBANB was consistently associated with better procedural and early postprocedural outcomes than topical EMLA or no pretreatment. Compared with the other strategies, UGBANB was associated with lower cumulative procedural pain burden, no requirement for intravenous rescue tramadol during application, higher treatment completion, and greater patient-reported tolerability and willingness to undergo repeat therapy. Although the observational design precludes causal conclusions regarding comparative effectiveness, the consistency of these associations across procedural, clinical, and patient-reported measures suggests that optimizing procedural analgesia may be an important component of successful capsaicin delivery. These observations are clinically relevant because capsaicin 8% patch therapy is delivered according to a standardized application protocol, with the recommended treatment duration forming an integral component of treatment delivery [12,15,16,21]. Premature patch removal because of intolerable discomfort may therefore prevent completion of the intended application and adversely affect the overall treatment experience. In the present cohort, all patients receiving UGBANB completed the planned application, whereas premature discontinuation occurred only in the EMLA and no pretreatment groups. Although this study was not designed to determine whether improved treatment completion translates into superior long-term analgesic outcomes, completion of the recommended application remains an important procedural endpoint in routine practice.

Overall, these findings suggest that optimized procedural analgesia may complement, rather than modify, the established pharmacological effects of capsaicin. Improving the application experience may facilitate successful delivery and acceptance of this evidence-based therapy while preserving its established role in the management of PDPN.

### 4.3. Ultrasound-Guided Bilateral Ankle Nerve Block as a Procedural Analgesic Strategy

The favorable procedural outcomes associated with UGBANB are anatomically and physiologically plausible. PDPN predominantly affects the distal lower extremities, while sensory innervation of the plantar and dorsal aspects of the foot is supplied by branches of the tibial, superficial peroneal, deep peroneal, sural, and saphenous nerves [4,29,30,31,37].. The ankle therefore provides an anatomically appropriate site for regional sensory blockade before capsaicin 8% patch application. Unlike topical EMLA, which produces predominantly superficial cutaneous analgesia [38,39], UGBANB temporarily interrupts afferent nociceptive transmission proximal to the capsaicin application site. This mechanistic distinction provides a plausible explanation for the lower cumulative procedural pain burden, reduced rescue analgesic requirements, and higher treatment completion observed with UGBANB in this cohort. However, because pretreatment allocation was non-randomized, these findings should be interpreted as associations rather than evidence that the nerve block itself caused the observed differences. Ultrasound-guided regional anesthesia is widely used within perioperative pain management strategies and continues to be evaluated prospectively across different anatomical and clinical settings [35] However, evidence specifically evaluating regional anesthesia as a procedural adjunct to capsaicin 8% patch therapy remains limited. UGBANB may also offer practical advantages for outpatient treatment when appropriate regional anesthesia expertise is available. Ultrasound guidance permits visualization of peripheral nerves, adjacent vascular structures, needle advancement, and local anesthetic spread, thereby improving block accuracy and procedural success compared with non-ultrasound-guided techniques [30,31,33,35]. No block-related complications were observed in the present cohort; however, the sample was too small to exclude uncommon adverse events. Previous studies support a generally favorable safety profile for ankle and peripheral nerve blocks when performed by appropriately trained clinicians [29,30,32,39,40]. Accordingly, UGBANB may represent a feasible procedural adjunct in appropriately selected patients undergoing capsaicin 8% patch therapy, although its broader implementation will depend on operator expertise, resource availability, procedural time, and confirmation of effectiveness and safety in prospective studies.

### 4.4. Mechanistic Interpretation

The pain trajectories observed in this study are biologically consistent with the established pharmacological mechanism of high-concentration capsaicin. Capsaicin selectively activates transient receptor potential vanilloid 1 (TRPV1) receptors expressed predominantly on peripheral nociceptive fibers, producing rapid cation influx and the characteristic burning sensation during application. Continued exposure is followed by reversible defunctionalization of nociceptive terminals and reduced peripheral nociceptive signaling, which contribute to the sustained analgesic effects of capsaicin therapy [11,13,14,17,18].These pharmacological effects are consistent with the temporal pain profile observed in this cohort, in which pain increased during the early phase of application, peaked at approximately 30 min, and subsequently declined. Repeated-measures analysis demonstrated a significant effect of time on procedural pain (F(6,534) = 17.36, *p* < 0.001; partial η^2^ = 0.163), while between-group comparisons at individual post-baseline assessments were also significant (all *p* < 0.001; Table 3). Although topical EMLA was associated with lower procedural pain than no pretreatment, clinically important discomfort persisted during application. This finding is biologically plausible because topical EMLA produces predominantly cutaneous analgesia of limited depth [47,48] and may therefore provide incomplete suppression of nociceptive input generated during prolonged high-concentration capsaicin exposure. In contrast, UGBANB interrupts afferent nociceptive transmission proximal to the application site, providing a plausible mechanistic explanation for the lower procedural pain burden and rescue analgesic requirements observed in this group [29,30,31].

A further observation was the lower pain intensity recorded in the UGBANB group during the early postprocedural period. These findings should be interpreted cautiously because residual sensory blockade from bupivacaine may have contributed to lower pain scores after patch removal [34] particularly during the earlier follow-up assessments. Differences in rescue analgesic exposure and other unmeasured factors may also have influenced postprocedural pain. Although temporary suppression of peripheral nociceptive input during capsaicin application could theoretically modify early nociceptive sensitization, the present observational study was not designed to evaluate this mechanism. Accordingly, the postprocedural pain findings should not be interpreted as evidence that UGBANB enhances the intrinsic or long-term analgesic efficacy of capsaicin. Prospective mechanistic studies incorporating standardized sensory testing and longer-term follow-up are needed to clarify these observations.

### 4.5. Clinical Implications for Outpatient Capsaicin Therapy

The present findings have important implications for the outpatient delivery of capsaicin 8% patch therapy. Although high-concentration capsaicin is an established treatment for localized peripheral neuropathic pain, application-related discomfort remains a recognized challenge that may affect treatment completion and patient acceptance [16,21,22,28,46]. The present findings suggest that optimizing procedural analgesia may facilitate the delivery of this evidence-based therapy without altering its established pharmacological mechanism of action. The observed benefits extended beyond procedural pain reduction. UGBANB was associated with no requirement for intravenous rescue tramadol during application, lower oral tramadol requirements during the subsequent 48 h, higher treatment completion, greater procedural tolerability, and greater willingness to undergo repeat therapy. Collectively, these findings suggest that effective procedural analgesia may improve multiple aspects of the treatment experience relevant to routine outpatient practice. The lower requirement for rescue opioid analgesia is also clinically relevant. Patients with PDPN may already receive multiple pharmacological treatments as part of neuropathic pain management] [1,2,3,8,9,10].. Minimizing additional opioid exposure during outpatient procedures is consistent with contemporary multimodal analgesia and opioid stewardship principles [23,24]. Although long-term opioid use was not evaluated, the absence of intravenous rescue tramadol in the UGBANB group suggests that regional anesthesia may reduce the requirement for supplemental opioid analgesia during capsaicin treatment.

Improved treatment completion and willingness to undergo repeat therapy may also have clinical implications because capsaicin 8% patch therapy can be repeated when clinically indicated to maintain symptom control [14,15,16,20].Although the present study did not evaluate long-term adherence or sustained analgesic outcomes, a more favorable treatment experience may increase patients’ acceptance of subsequent treatment cycles. However, willingness to undergo repeat treatment should not be interpreted as evidence of actual long-term adherence. Prospective longitudinal studies incorporating repeated treatment cycles, treatment persistence, and validated patient-reported quality-of-life outcomes are needed to evaluate this relationship [25,26,27].

### 4.6. Patient-Reported Outcomes and Treatment Acceptability

Patient-reported outcomes further support the clinical relevance of optimizing procedural analgesia during capsaicin 8% patch therapy. Compared with topical EMLA or no pretreatment, UGBANB was associated with greater procedural tolerability and willingness to undergo repeat therapy. These findings suggest that effective procedural analgesia may influence not only application-related pain but also patients’ overall experience and acceptance of treatment.

This is particularly relevant because capsaicin 8% patch therapy can be repeated when clinically indicated to maintain symptom control [14,15,16,20].Application-site burning is among the most commonly reported treatment-related adverse effects and may adversely affect the treatment experience and acceptance of subsequent applications [12,15,16,20,21,22,28]. Improving procedural tolerability may therefore represent an important component of patient-centered pain care [25,26] and could facilitate the integration of capsaicin therapy into routine neuropathic pain management.

Although long-term adherence was not evaluated, the greater procedural tolerability and willingness to undergo repeat therapy observed with UGBANB suggest that a more favorable treatment experience may support continued patient engagement with capsaicin therapy. However, willingness to undergo repeat treatment should not be interpreted as evidence of actual treatment persistence or long-term adherence. Prospective studies incorporating repeated treatment cycles, validated patient-reported outcome measures, treatment satisfaction, health-related quality of life, and longitudinal adherence outcomes are needed to determine whether improved procedural acceptability translates into sustained patient-centered or clinical benefits [25,26,27,49].

### 4.7. Comparison with Previous Research

The present findings are broadly consistent with the established evidence supporting high-concentration capsaicin for the management of localized peripheral neuropathic pain. Randomized controlled trials, longer-term studies, and real-world investigations have demonstrated clinically meaningful pain relief with an acceptable safety profile in appropriately selected patients with PDPN [11,12,13,14,15,16,19,20,28]. Clinical experience with capsaicin 8% patch therapy has also extended to other peripheral neuropathic pain conditions, including chemotherapy-induced peripheral neuropathy [50]. However, previous research has focused predominantly on analgesic efficacy, durability of response, and longer-term safety, whereas comparatively less attention has been directed toward strategies for improving tolerability during patch application.

Application-related burning pain remains an important limitation of capsaicin 8% patch therapy and may adversely affect treatment completion and acceptance of subsequent applications [16,21,22,28,46].Various supportive approaches have been explored to improve the procedural experience, including topical analgesic strategies and, more recently, non-pharmacological adjuncts such as virtual reality during capsaicin application [21,22,51].However, evidence specifically evaluating regional anesthesia as a procedural adjunct remains limited. The present study extends this literature by demonstrating an association between an anatomy-specific regional anesthesia technique and lower procedural pain burden, reduced rescue analgesic requirements, higher treatment completion, and greater patient-reported acceptability compared with topical EMLA or no pretreatment. Although the observational design precludes causal conclusions regarding comparative effectiveness, these findings suggest that procedural analgesia warrants greater consideration as a component of successful capsaicin treatment delivery. These observations also complement our previous retrospective cohort study evaluating ultrasound-guided thoracic paravertebral block as an adjunct to capsaicin 8% patch therapy for thoracic postherpetic neuralgia, which similarly examined regional anesthesia as a strategy for improving procedural tolerability [36] Despite differences in the anatomical target and underlying neuropathic pain condition, both studies identified associations between regional anesthesia-assisted treatment and improved procedural tolerability, lower rescue analgesic requirements, and greater treatment completion. Together, these observations support the hypothesis that temporary interruption of nociceptive transmission proximal to the capsaicin application site may facilitate treatment delivery without implying enhancement of capsaicin’s intrinsic pharmacological efficacy.

Collectively, these observations raise the hypothesis that procedural tolerability during capsaicin 8% patch therapy may be improved by selecting an anatomically appropriate sensory block matched to the distribution of neuropathic pain, rather than by any single regional anesthesia technique. This concept provides a potential framework for investigating procedural optimization across different peripheral neuropathic pain conditions. However, comparisons between the present study and previous reports should be interpreted cautiously because of differences in study design, patient populations, anatomical targets, pretreatment protocols, outcome measures, and follow-up duration. Prospective multicenter studies are therefore needed to determine whether anatomy-directed regional anesthesia can reproducibly improve capsaicin treatment delivery across different neuropathic pain conditions. These findings should be regarded as complementary to, rather than as replacing or modifying, the established evidence supporting the pharmacological efficacy of capsaicin 8% patch therapy.

### 4.8. Safety Considerations

Alongside the favorable procedural outcomes, no evidence of compromised short-term safety was observed. Transient localized erythema occurred in all patients, regardless of pretreatment group, and resolved spontaneously without additional intervention. This finding is consistent with the expected application-site reactions associated with high-concentration capsaicin reported in clinical trials and real-world studies]. [12,15,16,19,20,21,22,28]. Importantly, no serious treatment-related adverse events or block-related complications were identified during the available follow-up period. No cases of blistering, skin ulceration, secondary infection, persistent sensory deficit, delayed neurological complication, treatment-related hospitalization, vascular puncture, or local anesthetic systemic toxicity (LAST) were documented. These observations are consistent with the generally favorable safety profile reported for ultrasound-guided ankle and peripheral nerve blocks when performed using appropriate techniques [29,30,31,32,33,34,35,40].

A potential concern when incorporating lower-limb peripheral nerve blocks into ambulatory treatment pathways is transient sensory impairment and its potential effect on safe ambulation, particularly in patients with diabetic peripheral neuropathy who may already have impaired protective sensation and increased susceptibility to balance impairment and falls [39,41]. In the present cohort, no prolonged numbness, clinically significant motor weakness, delayed recovery, falls, or delayed discharge were documented. Although reassuring, these observations should not be interpreted as establishing the absence of risk. They support the feasibility of short-term outpatient management within a standardized pathway incorporating ultrasound guidance, postprocedural assessment, patient education, and precautions during temporary sensory impairment [30,39,40].

Nevertheless, these safety findings should be interpreted cautiously. The modest sample size, particularly the 30 patients who received UGBANB, limited the ability to detect uncommon block-related adverse events, and all procedures were performed at a single tertiary referral center by clinicians experienced in regional anesthesia. Accordingly, the absence of observed complications should not be interpreted as evidence of zero risk. Larger prospective multicenter studies are needed to provide more precise estimates of uncommon adverse events and to evaluate sensory and motor recovery, ambulation, falls, inadvertent foot injury, and longer-term neurological safety following regional anesthesia-assisted capsaicin therapy.

### 4.9. Strengths and Clinical Relevance

Several strengths should be acknowledged. To our knowledge, this is the first comparative study to evaluate UGBANB as a procedural analgesic adjunct to capsaicin 8% patch therapy specifically in patients with PDPN. Rather than re-examining the established analgesic efficacy of capsaicin, the study addressed the comparatively underexplored challenge of improving procedural tolerability and treatment delivery.

Another strength was the use of a standardized institutional treatment pathway incorporating neurologist-confirmed diagnosis, predefined eligibility criteria, uniform capsaicin application procedures, structured pain assessments, standardized rescue analgesia criteria, and consistent follow-up. Importantly, all participants underwent the same capsaicin treatment protocol, outcome assessment schedule, and follow-up procedures irrespective of pretreatment allocation, thereby minimizing procedural variability unrelated to the pretreatment strategy.

This study also incorporated a multidimensional assessment of clinically relevant procedural outcomes. Rather than relying solely on isolated pain-intensity measurements, cumulative procedural pain burden was quantified using AUC-NPRS, which integrates serial pain measurements over time and provides a more comprehensive representation of the procedural pain experience [27,42,43]. Complementary outcomes included rescue analgesic requirements, treatment completion, early postprocedural pain, patient-reported procedural tolerability, willingness to undergo repeat treatment, and predefined safety outcomes. The consistency of the observed associations across these complementary domains strengthens the overall interpretation of the findings. From a clinical perspective, the study addresses a practical challenge in the outpatient delivery of capsaicin 8% patch therapy: managing application-related discomfort while facilitating completion and patient acceptance. The findings suggest that anatomy-specific regional anesthesia may represent a useful procedural strategy for selected patients, particularly when substantial application-related discomfort is anticipated or conventional pretreatment provides inadequate tolerability. However, implementation requires appropriate regional anesthesia expertise, ultrasound availability, additional procedural time, and structured post-block monitoring. Accordingly, the present findings should be considered preliminary and hypothesis-generating rather than sufficient to support routine UGBANB for all patients undergoing capsaicin therapy.Prospective randomized studies of ultrasound-guided regional anesthesia are increasingly being used to evaluate procedural effectiveness across different clinical settings [52]. Accordingly, multicenter randomized studies are needed to define which patients are most likely to benefit from UGBANB and to determine the comparative effectiveness, safety, resource requirements, and cost-effectiveness of this approach.

### 4.10. Limitations

Several limitations should be considered when interpreting these findings. First, the retrospective observational design precludes causal inference and is susceptible to selection bias, confounding by indication, and residual confounding despite comparable measured baseline characteristics across the three groups. Pretreatment allocation reflected routine clinical practice rather than randomization and was influenced by clinician judgment, patient preference, and operator availability. Consequently, unmeasured factors such as anticipated procedural tolerability, anxiety, previous treatment experience, or clinician preference may have influenced group assignment and contributed to the magnitude of the observed between-group differences. The findings should therefore be interpreted as associations rather than evidence of comparative effectiveness.

Second, cumulative procedural pain burden was calculated from observed NPRS measurements without extrapolation beyond the final recorded assessment in patients who discontinued capsaicin application prematurely. Because discontinuation occurred predominantly in the no pretreatment and EMLA groups, this approach may have underestimated cumulative pain burden in these groups and thereby attenuated the observed between-group differences. Sensitivity analyses using post-discontinuation imputation were not performed because they would have required unverifiable assumptions regarding unobserved pain trajectories. Future prospective studies should incorporate predefined approaches for handling early discontinuation.

Third, pain assessments were obtained during routine clinical care, and personnel recording NPRS scores were aware of the pretreatment strategy because blinding was not practically feasible. Detection or observer bias therefore cannot be excluded. Similarly, procedural tolerability and willingness to undergo repeat treatment were derived from standardized routine clinical documentation rather than validated patient-reported outcome measures. Although these outcomes provide clinically relevant information regarding treatment acceptability, future studies should incorporate validated measures of treatment satisfaction and patient experience.

Fourth, PDPN diagnosis was based on specialist clinical assessment and contemporaneous neurological documentation rather than systematic use of a validated neuropathic pain screening instrument such as DN4 or painDETECT. Although diagnoses were independently confirmed by a consultant neurologist and a fellowship-trained Pain Medicine consultant, the absence of a uniformly applied validated diagnostic instrument should be considered when interpreting diagnostic standardization.

Fifth, the study was conducted at a single tertiary referral center and included a relatively modest sample of 90 patients, of whom only 30 underwent UGBANB. This limits generalizability and the precision of estimates for uncommon adverse events. All nerve blocks were also performed by experienced clinicians using a standardized ultrasound-guided protocol; therefore, the observed procedural outcomes may not be directly reproducible in settings with different levels of operator expertise, ultrasound availability, or institutional resources. The absence of observed block-related complications should not be interpreted as evidence of zero risk.

Sixth, the study focused on procedural and early postprocedural outcomes. Lower pain scores during the early postprocedural period may have been influenced by residual bupivacaine-mediated sensory blockade, differences in rescue analgesic exposure, or other unmeasured factors and should not be interpreted as evidence that UGBANB enhances the intrinsic or long-term analgesic efficacy of capsaicin. Subsequent treatment cycles, actual long-term adherence, sustained analgesic efficacy, functional outcomes, health-related quality of life, healthcare utilization, and cost-effectiveness were not evaluated.

Despite these limitations, the study provides preliminary evidence that anatomy-specific regional anesthesia may represent a promising strategy for improving the procedural tolerability and delivery of capsaicin 8% patch therapy. Prospective multicenter randomized controlled trials incorporating standardized rescue analgesia, blinded outcome assessment where feasible, validated patient-reported measures, predefined handling of treatment discontinuation, longer safety surveillance, and longitudinal follow-up are needed to confirm these associations and determine whether improved procedural tolerability translates into better treatment adherence, clinical outcomes, health-related quality of life, and cost-effectiveness.

## 5. Conclusions

In this retrospective comparative cohort study, UGBANB was associated with consistently better procedural outcomes than topical EMLA or no pretreatment during capsaicin 8% patch therapy for PDPN. UGBANB was associated with lower cumulative procedural pain burden, reduced rescue tramadol requirements, higher treatment completion, greater procedural tolerability, and greater willingness to undergo repeat therapy, without an apparent increase in short-term treatment- or block-related complications.

These findings suggest that effective procedural analgesia may represent an important component of successful capsaicin treatment delivery. By improving the application experience without modifying the established pharmacological action of capsaicin, anatomy-specific regional anesthesia may facilitate treatment completion and patient acceptance in appropriately selected patients undergoing capsaicin therapy.

Because of the retrospective, non-randomized, single-center design, these associations should not be interpreted as causal or as evidence of superior long-term analgesic efficacy. Prospective multicenter randomized controlled trials are needed to confirm these findings and determine whether improved procedural tolerability translates into sustained benefits in treatment adherence, long-term clinical outcomes, health-related quality of life, safety, and cost-effectiveness.

## Figures and Tables

**Table 1:** Baseline Demographic and Clinical Characteristics of Study Population.

Characteristic	No Pretreatment (n = 30)	Topical EMLA (n = 30)	Ultrasound-Guided Bilateral Ankle Nerve Block (n = 30)	*p* Value
**Demographic characteristics**				
Age, years, mean ± SD	62.1 ± 8.3	61.7 ± 8.8	63.0 ± 7.9	0.83
Male sex, n (%)	18 (60.0)	20 (66.7)	18 (60.0)	0.83
Body mass index, kg/m^2^, mean ± SD	29.4 ± 4.1	30.0 ± 3.9	28.9 ± 4.3	0.58
Current smoking, n (%)	4 (13.3)	7 (23.3)	7 (23.3)	0.51
**Clinical characteristics**				
Type 2 diabetes mellitus, n (%)	30 (100)	30 (100)	30 (100)	NA
Diabetes duration, years, mean ± SD	12.7 ± 4.4	13.1 ± 4.9	12.8 ± 4.5	0.94
PDPN duration, years, mean ± SD	3.8 ± 1.6	4.1 ± 1.7	3.9 ± 1.5	0.79
HbA1c, %, mean ± SD	7.8 ± 1.1	7.6 ± 1.2	7.9 ± 1.1	0.58
Baseline NPRS score, mean ± SD	6.9 ± 1.1	7.0 ± 1.2	6.8 ± 1.1	0.79
Bilateral foot involvement, n (%)	28 (93.3)	29 (96.7)	28 (93.3)	0.81
Previous capsaicin 8% patch therapy, n (%)	0 (0)	0 (0)	0 (0)	NA
**Medical comorbidities**				
Hypertension, n (%)	20 (66.7)	17 (56.7)	22 (73.3)	0.42
Chronic kidney disease (eGFR < 30 mL/min/1.73 m^2^), n (%)	5 (16.7)	5 (16.7)	7 (23.3)	0.79
Anxiety disorder, n (%)	3 (10.0)	6 (20.0)	5 (16.7)	0.55
Depression, n (%)	6 (20.0)	8 (26.7)	12 (40.0)	0.22
**Baseline pharmacological therapy**				
Pregabalin use, n (%)	24 (80.0)	23 (76.7)	25 (83.3)	0.81
Gabapentin use, n (%)	8 (26.7)	10 (33.3)	9 (30.0)	0.88
Amitriptyline use, n (%)	10 (33.3)	11 (36.7)	9 (30.0)	0.85
SSRI use, n (%)	6 (20.0)	8 (26.7)	12 (40.0)	0.22
SNRI use, n (%)	3 (10.0)	6 (20.0)	5 (16.7)	0.55

Abbreviations: EMLA, eutectic mixture of lidocaine and prilocaine; eGFR, estimated glomerular filtration rate; HbA1c, glycated hemoglobin; NPRS, Numeric Pain Rating Scale; PDPN, painful diabetic peripheral neuropathy; SD, standard deviation; SNRI, serotonin–norepinephrine reuptake inhibitor; SSRI, selective serotonin reuptake inhibitor; NA: Not applicable because no participant in the ultrasound-guided bilateral ankle nerve block group required intravenous rescue analgesia. Footnote: Continuous variables are presented as the mean ± standard deviation (SD) and were compared using one-way analysis of variance (ANOVA). Categorical variables are presented as number (percentage) and were compared using the chi-square test or Fisher’s exact test, as appropriate. *p* values are two-sided. All included patients were receiving guideline-directed management for PDPN before referral to the Pain Medicine Clinic.

**Table 2:** Cumulative Procedural Pain Burden During Standardized 60-min Capsaicin 8% Patch Application According to Pretreatment Strategy.

Pretreatment Strategy	n	Mean AUC-NPRS (NPRS·min) ± SD	Mean Difference (95% CI) *	Adjusted *p* Value	Cohen’s *d*
No pretreatment	30	481.5 ± 39.2	Reference	—	—
Topical EMLA pretreatment	30	429.2 ± 36.0	−52.3 (−75.7 to −29.0)	<0.001	1.39
Ultrasound-guided bilateral ankle nerve block	30	256.3 ± 38.4	−225.2 (−248.5 to −201.8) †	<0.001	5.8

Overall comparison: One-way ANOVA: *F* = 289.8; *p* < 0.001; η^2^ = 0.87. Abbreviations: AUC-NPRS, area under the Numeric Pain Rating Scale–time curve; ANOVA, analysis of variance; CI, confidence interval; EMLA, eutectic mixture of lidocaine and prilocaine; SD, standard deviation. Footnotes: Data are presented as mean ± standard deviation (SD). Overall between-group differences were evaluated using one-way analysis of variance (ANOVA) followed by Tukey’s honestly significant difference (HSD) post hoc test. * Mean differences are presented relative to the no pretreatment group. † Comparison between the ultrasound-guided bilateral ankle nerve block and topical EMLA pretreatment: mean difference −172.8 NPRS·min (95% CI, −196.2 to −149.5); adjusted *p* < 0.001; Cohen’s *d* = 4.64.

**Table 3:** Procedural Pain Intensity During the Standardized 60-min Capsaicin 8% Patch Application According to Pretreatment Strategy.

Time Point	No Pretreatment (n = 30) Mean ± SD	Topical EMLA Pretreatment (n = 30) Mean ± SD	Ultrasound-Guided Bilateral Ankle Nerve Block (n = 30) Mean ± SD	Overall *p* Value †
Baseline	6.9 ± 1.1	7.0 ± 1.2	6.8 ± 1.1	0.79
10 min	7.6 ± 0.9	7.0 ± 1.0	3.6 ± 0.8	<0.001
20 min	8.2 ± 0.9	7.3 ± 1.0	4.2 ± 0.9	<0.001
30 min	8.5 ± 0.8	7.6 ± 1.0	4.5 ± 0.9	<0.001
40 min	8.3 ± 0.9	7.4 ± 0.9	4.3 ± 0.8	<0.001
50 min	8.0 ± 1.0	7.1 ± 1.0	4.0 ± 0.8	<0.001
60 min	7.6 ± 1.0	6.8 ± 1.0	3.8 ± 0.8	<0.001

Abbreviations: EMLA, eutectic mixture of lidocaine and prilocaine; SD, standard deviation. Footnotes: Data are presented as the mean ± standard deviation (SD). † Overall between-group comparisons at each assessment time point were performed using one-way analysis of variance (ANOVA). Temporal changes in procedural pain intensity were further evaluated using repeated-measures ANOVA. Significant effects of time were observed (F(6,534) = 17.36, *p* < 0.001, partial η^2^ = 0.163). Detailed repeated-measures ANOVA results and Bonferroni-adjusted pairwise comparisons are provided in Supplementary Table S1.

**Table 4:** Rescue Analgesia Requirements During and After Standardized Capsaicin 8% Patch Application According to Pretreatment Strategy.

Outcome	No Pretreatment (n = 30)	Topical EMLA Pretreatment (n = 30)	Ultrasound-Guided Bilateral Ankle Nerve Block (n = 30)	Effect Estimate	*p* Value
Intravenous tramadol during treatment					
Participants requiring rescue analgesia, n (%)	30 (100.0)	18 (60.0)	0 (0.0)	EMLA vs. No pretreatment: RR = 0.60 (95% CI, 0.43–0.84); Nerve block vs. No pretreatment: RR = 0.03 (95% CI, 0.002–0.41)	<0.001
Cumulative intravenous dose, mg, mean ± SD †	87.5 ± 14.8	63.9 ± 13.6	0	EMLA vs. No pretreatment: MD = −23.6 mg (95% CI, −33.8 to −13.4)	<0.001
Oral tramadol within 48 h					
Participants requiring rescue analgesia, n (%)	30 (100.0)	18 (60.0)	6 (20.0)	EMLA vs. No pretreatment: RR = 0.60 (95% CI, 0.43–0.84); Nerve block vs. No pretreatment: RR = 0.20 (95% CI, 0.09–0.47)	<0.001
Cumulative oral dose, mg, mean ± SD ‡	228.3 ± 46.2	164.7 ± 50.8	91.7 ± 18.5	EMLA vs. No pretreatment: MD = −63.6 mg (95% CI, −91.4 to −35.8); Nerve block vs. No pretreatment: MD = −136.6 mg (95% CI, −162.1 to −111.1)	<0.001

Abbreviations: CI, confidence interval; EMLA, eutectic mixture of lidocaine and prilocaine; MD, mean difference; RR, risk ratio; SD, standard deviation. Footnotes: Data are presented as a number (percentage) or the mean ± standard deviation (SD). † Calculated among participants who received intravenous tramadol during capsaicin application. ‡ Calculated among participants who required oral tramadol during the first 48 h after treatment. Risk ratios (RRs) and mean differences (MDs) are presented relative to the no pretreatment group.

**Table 5:** Postprocedural Pain Intensity Following Standardized Capsaicin 8% Patch Application.

Time After Treatment	Mean NPRS ± SD
8 h	4.58 ± 1.34
24 h	3.74 ± 1.11
48 h	3.17 ± 1.00

Abbreviations: NPRS, Numeric Pain Rating Scale; SD, standard deviation. Footnotes: Data are presented as the mean ± standard deviation (SD). Changes in postprocedural pain intensity over time were evaluated using repeated-measures analysis of variance (ANOVA). A significant effect of time was observed (F(2,178) = 231.36, *p* < 0.001; partial η^2^ = 0.722). Bonferroni-adjusted pairwise comparisons demonstrated significant reductions in pain intensity between 8 and 24 h, 24 and 48 h, and 8 and 48 h (all *p* < 0.001). Detailed statistical results are provided in Supplementary Table S1.

**Table 6:** Treatment Completion During Standardized Capsaicin 8% Patch Application According to Pretreatment Strategy.

Outcome	No Pretreatment (n = 30)	Topical EMLA Pretreatment (n = 30)	Ultrasound-Guided Bilateral Ankle Nerve Block (n = 30)	Effect Estimate vs. No Pretreatment	*p* Value
Completed planned 60 min application, n (%)	22 (73.3)	25 (83.3)	30 (100.0)	EMLA: RR = 1.14 (95% CI, 0.89–1.46); Nerve block: RR = 1.36 (95% CI, 1.08–1.71)	0.018
Premature treatment discontinuation, n (%)	8 (26.7)	5 (16.7)	0 (0.0)	—	0.018
Application duration among participants with premature discontinuation, median (IQR), min †	36 (33–39)	39 (35–42)	NA	—	—

Abbreviations: CI, confidence interval; EMLA, eutectic mixture of lidocaine and prilocaine; IQR, interquartile range; RR, risk ratio. Footnotes: Data are presented as a number (percentage) or the median (interquartile range [IQR]), as appropriate. Categorical variables were compared using Fisher’s exact test, and risk ratios (RRs) with 95% confidence intervals (CIs) were calculated using the no pretreatment group as the reference. † Calculated only among participants who discontinued treatment before completion of the planned 60 min capsaicin 8% patch application. NA: Not applicable because no participant in the ultrasound-guided bilateral ankle nerve block group discontinued treatment prematurely.

**Table 7:** Patient-Reported Outcomes Following Standardized Capsaicin 8% Patch Application According to Pretreatment Strategy.

Outcome	No Pretreatment (n = 30)	Topical EMLA Pretreatment (n = 30)	Ultrasound-Guided Bilateral Ankle Nerve Block (n = 30)	Effect Estimate vs. No Pretreatment	*p* Value
Procedural tolerability, n (%)					
Well tolerated	0 (0.0)	0 (0.0)	27 (90.0)	—	
Moderate discomfort	0 (0.0)	5 (16.7)	3 (10.0)	—	
Severe procedural burning pain	30 (100.0)	25 (83.3)	0 (0.0)	—	<0.001 †
Willingness to undergo repeat capsaicin 8% patch therapy, n (%)	10 (33.3)	18 (60.0)	30 (100.0)	EMLA: RR = 1.80 (95% CI, 1.01–3.22)Nerve block: RR = 3.00 (95% CI, 1.76–5.12)	<0.001

Abbreviations: CI, confidence interval; EMLA, eutectic mixture of lidocaine and prilocaine; RR, risk ratio. Footnotes: Data are presented as number (percentage). † Overall comparison of the distribution of procedural tolerability categories among the three pretreatment groups using Fisher’s exact test. Risk ratios (RRs) and 95% confidence intervals (CIs) for willingness to undergo repeat capsaicin 8% patch therapy were calculated using the no pretreatment group as the reference category.

**Table 8:** Safety Outcomes Following Standardized Capsaicin 8% Patch Application According to Pretreatment Strategy.

Safety Outcome	No Pretreatment (n = 30)	Topical EMLA Pretreatment (n = 30)	Ultrasound-Guided Bilateral Ankle Nerve Block (n = 30)	Overall (N = 90)
Capsaicin-related adverse events				
Transient localized erythema	30 (100.0)	30 (100.0)	30 (100.0)	90 (100.0)
Blistering or skin ulceration	0 (0.0)	0 (0.0)	0 (0.0)	0 (0.0)
Secondary infection	0 (0.0)	0 (0.0)	0 (0.0)	0 (0.0)
Systemic adverse events	0 (0.0)	0 (0.0)	0 (0.0)	0 (0.0)
Persistent sensory deficits	0 (0.0)	0 (0.0)	0 (0.0)	0 (0.0)
Delayed neurological complications	0 (0.0)	0 (0.0)	0 (0.0)	0 (0.0)
Treatment-related hospitalization	0 (0.0)	0 (0.0)	0 (0.0)	0 (0.0)
Ultrasound-guided ankle nerve block-related adverse events *				
Local anesthetic systemic toxicity (LAST)	—	—	0 (0.0)	0 (0.0)
Vascular puncture	—	—	0 (0.0)	0 (0.0)
Block-related complications	—	—	0 (0.0)	0 (0.0)

Footnotes: Data are presented as a number (percentage). * Block-related adverse events were evaluated only among participants who underwent ultrasound-guided bilateral ankle nerve block. Localized erythema represented the expected transient local cutaneous reaction associated with high-concentration capsaicin therapy and resolved without additional treatment. No formal between-group statistical comparisons were performed because no serious adverse events were observed in any study group.

## Data Availability

The data presented in this study are available from the corresponding author upon reasonable request. Although the dataset does not contain direct participant identifiers, it is not publicly available due to ethical and privacy considerations related to the potential risk of participant re-identification.

## Supplementary Materials

The following supporting information can be downloaded at: https://www.mdpi.com/article/10.3390/healthcare14162563/s1, Table S1: Repeated-Measures Analyses of Procedural and Early Postprocedural Pain During Capsaicin 8% Patch Therapy.

## Abbreviations

AUC-NPRS	Area under the Numeric Pain Rating Scale–time curve
CI	Confidence interval
EMLA	Eutectic mixture of lidocaine/prilocaine
EMR	Electronic medical record
LAST	Local anesthetic systemic toxicity
NPRS	Numeric Pain Rating Scale
PDPN	Painful diabetic peripheral neuropathy
PROMs	Patient-reported outcome measures
RCTs	Randomized controlled trials
RR	Risk ratio
SD-	Standard deviation
TRPV1	Transient receptor potential vanilloid 1
UGBANB	Ultrasound-guided bilateral ankle nerve block
