# Supplementary material for: Procedural Optimization of Capsaicin 8% Patch Therapy Using Ultrasound-Guided Bilateral Ankle Nerve Block in Painful Diabetic Peripheral Neuropathy: A Retrospective Comparative Cohort Study
Source: Healthcare (Basel). 2026 Aug 16;14(16):2563. doi: 10.3390/healthcare14162563 (PMC13511762; doi:10.3390/healthcare14162563)
Supplement: Supplementary file 1 [file healthcare-14-02563-s001.zip › healthcare-4405345-supplementary.pdf]

**Table S1.** Repeated-Measures Analyses of Procedural and Early Postprocedural Pain During Capsaicin 8% Patch Therapy

*(A) Overall Repeated-Measures ANOVA for Procedural Pain During the Standardized 60-Minute Capsaicin Ap-plication*

| Effect | F | df | P value | Partial $\eta^2$ |
|---|---|---|---|---|
| Time | **17.36** | **6, 534** | **<0.001** | **0.163** |

*(B) Bonferroni-Adjusted Pairwise Comparisons of Procedural Pain Scores*

| Comparison | Adjusted P value | Interpretation |
|---|---|---|
| Baseline vs 10 min | 0.0033 | Significant |
| Baseline vs 20 min | 1 | Not significant |
| Baseline vs 30 min | 1 | Not significant |
| Baseline vs 40 min | 1 | Not significant |
| Baseline vs 50 min | 0.344 | Not significant |
| Baseline vs 60 min | 0.0016 | Significant |
| 10 vs 20 min | <0.0001 | Significant |
| 10 vs 30 min | <0.0001 | Significant |
| 10 vs 40 min | <0.0001 | Significant |
| 10 vs 50 min | 0.0011 | Significant |
| 10 vs 60 min | 1 | Not significant |
| 20 vs 30 min | <0.0001 | Significant |
| 20 vs 40 min | 1 | Not significant |
| 20 vs 50 min | 1 | Not significant |
| 20 vs 60 min | <0.0001 | Significant |
| 30 vs 40 min | <0.0001 | Significant |
| 30 vs 50 min | <0.0001 | Significant |
| 30 vs 60 min | <0.0001 | Significant |
| 40 vs 50 min | 0.22 | Not significant |
| 40 vs 60 min | <0.0001 | Significant |
| 50 vs 60 min | 0.0057 | Significant |

*(C) Overall Repeated-Measures ANOVA for Early Postprocedural Pain*

| Effect | F | df | P value | Partial $\eta^2$ |
|---|---|---|---|---|
| Time | 231.36 | 2, 178 | <0.001 | 0.722 |

*(D) Bonferroni-Adjusted Pairwise Comparisons of Early Postprocedural Pain*

| Comparison | Mean Difference | t (df) | Adjusted P value | Interpretation |
|---|---|---|---|---|
| 8 h vs 24 h | −0.84 | 12.24 (89) | <0.001 | Significant |
| 24 h vs 48 h | −0.57 | 10.38 (89) | <0.001 | Significant |
| 8 h vs 48 h | −1.41 | 19.32 (89) | <0.001 | Significant |

Abbreviations: ANOVA, analysis of variance; EMLA, eutectic mixture of lidocaine and prilocaine; NPRS, Numeric Pain Rating Scale.

Procedural pain was assessed at baseline and every 10 minutes during the standardized 60-minute capsaicin 8% patch application. Early postprocedural pain was assessed at 8, 24, and 48 hours after treatment. Repeated-measures analyses were performed using repeated-measures ANOVA. Mauchly's test was used to assess the assumption of sphericity, and the Greenhouse–Geisser correction was applied when appropriate. Bonferroni adjustment was used for all pairwise comparisons. Effect sizes are reported as partial eta squared (partial $\eta^2$).

1. Time effect for procedural pain: **F(6, 534) = 17.36**
2. Time effect for postprocedural pain: **F(2, 178) = 231.36**
3. Bonferroni pairwise comparisons
